# The Impact of Subsyndromal Bipolar Symptoms on Patient’s Functionality and Quality of Life

**DOI:** 10.3389/fpsyt.2020.00510

**Published:** 2020-06-12

**Authors:** Heinz Grunze, Christoph Born

**Affiliations:** Psychiatrie Schwäbisch Hall and PMU Nuremberg, Nuremberg, Germany

**Keywords:** bipolar disorder, subsyndromal, depression, mania, functionality, quality of life

## Abstract

Subsyndromal symptoms have rarely been in the focus of bipolar research. This may be, in part, due to the fact that there is neither a uniform definition nor do they constitute an indication of regulatory and commercial interest. Nevertheless, they do have a decisive impact on the long-term course of bipolar disorder (BD), and the degree of functionality and quality of life (QoL) is more likely determined by their presence or absence than by acute episodes. Summarizing the literature an estimated 20–50% of patients suffer inter-episodically or chronically from subsyndromal BD. The most prominent symptoms that interfere with functionality are subsyndromal depression, disturbances of sleep, and perceived cognitive impairment, whereas anxiety negatively impacts on QoL. In the absence of evidence-based pharmacological treatments for subsyndromal BD, clinical practice adopts guidelines designed for treatment-resistant full-blown episodes of BD, supplemented by cognitive-behavioral, family focused or social-rhythm–based psychotherapies.

## Introduction

**Figure 1:**
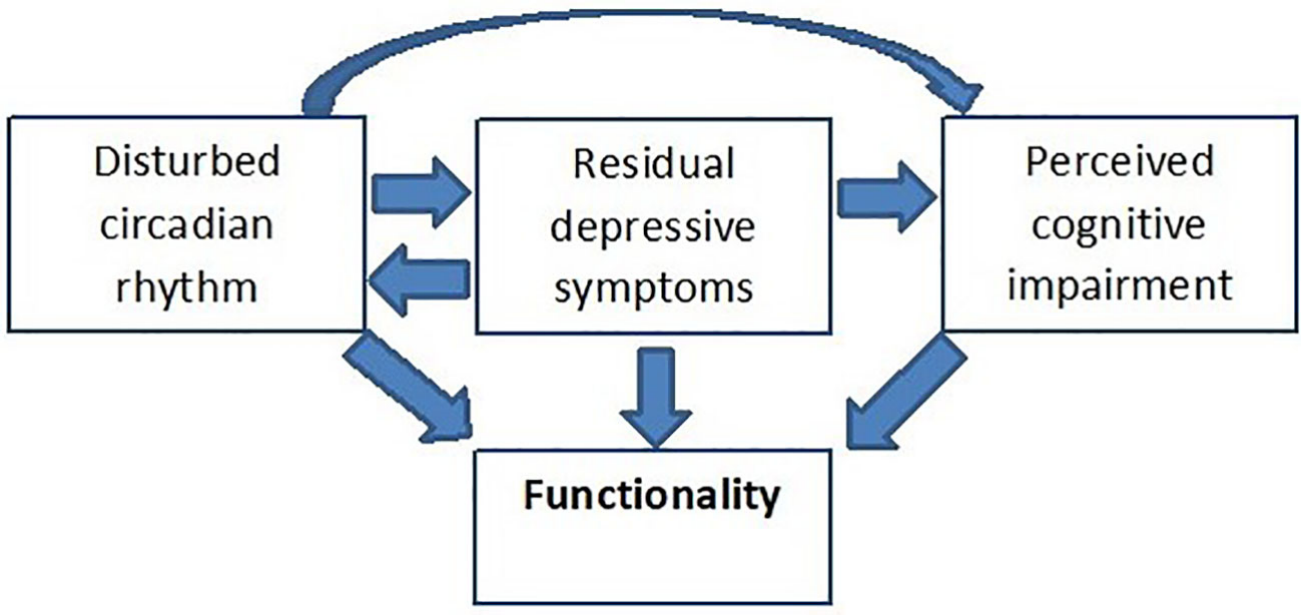
The trias of SSBD, their interaction and impact on functionality. A hypothetical model adapted from (5). Applying a structural equation model (SEM), Samalin et al. (5) demonstrated a significant direct effect of both residual depressive symptoms and perceived cognitive impairment on functionality. In addition, residual depressive symptoms also have a direct effect on perceived cognitive impairment but no significant indirect effect on functioning *via* perceived cognitive impairment. The direct pathway between sleep disturbances and functioning was not significant, however, the SEM confirmed the presence of a moderate indirect effect of sleep disturbances on functioning *via* residual depressive symptoms and perceived cognitive impairments.

Reality has taught us that Kraepelin’s assumption of full recovery as a decisive distinction between manic-depressive illness and dementia praecox does not hold true in a fair proportion of bipolar patients. Five years after onset of bipolar disorder (BD), at least 13% of patients suffer from a chronic course without remission (1). Persisting subsyndromal symptoms of BD (SSBD) impact on patients functionality and quality of life (QoL), and put them on elevated risk of relapse (2) and an overall more detrimental course of illness with longer duration of illness episodes and more lifetime psychotic symptoms (3). Adapting the dimensional view of BD as proposed by van Os and Kapur (4) subsyndromal symptoms are not necessarily restricted to mood, but may also persist in the domains of cognition and, if present during an acute episode, positive, and negative symptoms. However, little is known about the effects of persisting psychotic symptoms in bipolar patients, the bulk of data points toward subthreshold depression, impaired cognition and disturbed circadian rhythm as the most relevant SSBD (**Figure 1**) (5–8). Both subsyndromal depression and impaired cognition appear to act directly and independently on functionality (5, 8), whereas there is only a moderate indirect effect of sleep disturbances on functioning mediated *via* residual depressive symptoms and perceived cognitive impairments (5). Whereas subsyndromal depressive symptoms might be a crucial driver of psychosocial disability in all age groups, subsyndromal mania may be more abundant in old age bipolar patients than in younger patients (9).

SSBD is a topic that has been neglected for a long time in bipolar research. This may be, in part, due to the fact that there is neither a uniform definition and understanding what constitutes SSBD, nor does SSBD constitute an indication eligible for a marketing claim. However, with recent research on the nature, impact and mostly psychological interventions it appears timely to summarize our—still limited—knowledge.

**Figure 2:**
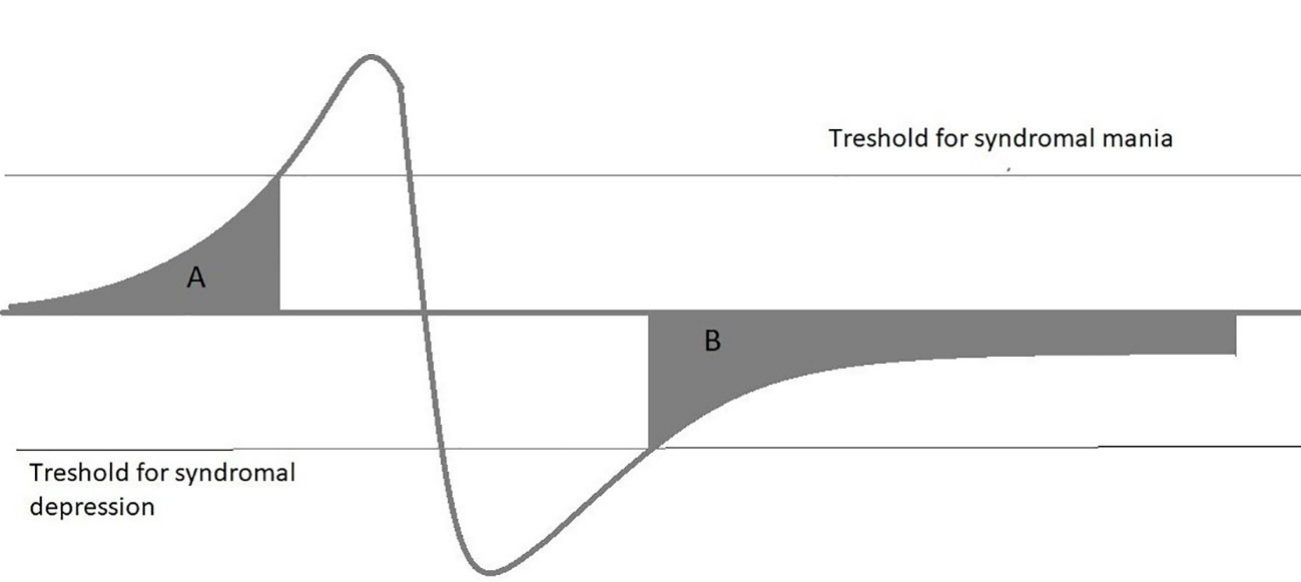
Subsyndromal symptoms in BD may occur as integral part of some bipolar spectrum disorders, e.g., cyclothymia, where mood swings do not fulfil full syndromal criteria, as precursors of a full-blown episode **(A)** or as residual symptoms after either a manic or depressive episode, sometimes with chronicity **(B)**.

This review focuses on three topics: Definition and frequency of SSBD, Impact of SSBD on functionality and QoL, and pharmacological and psychological therapies applied in SSBD. The review is not meant to be a complete and systematic review of the topic, it summarizes selected findings on SSBD and its treatment to support clinicians in identifying SSBD and guiding treatment selection. The review is based on a PubMed search covering published articles between 1970 and 2019 with the search terms “Bipolar disorder”, “mania”, “bipolar depression”, combined with either “subsyndromal”, “minor”, and “chronic”. Papers selected for this review are the authors’ subjective choice based on perceived novelty and general interest of the findings reported. In addition, reviewers of this article added valuable suggestions on further reports that deserve inclusion. Subsyndromal symptoms in BD may occur as integral part of some bipolar spectrum disorders, e.g., cyclothymia, where mood swings do not satisfy full syndromal criteria, as precursors of a full-blown episode or as residual symptoms after either a manic or depressive episode, sometimes with chronicity (**Figure 2**). Subsyndromal symptoms are observable in a majority of patients during the prodrome of a new manic or depressive episode, with manic episodes having a longer prodrome than depression (10–12). In adolescents, prodromal syndromes may precede a first episodes for years, and their duration and extent might predict later service usage (13). Besides mood and subthreshold psychotic symptoms, anxiety disorders have been identified as a frequent prodromal bipolar symptom (14). The focus of this article, however, is on subsyndromal symptoms in the aftermath of an episode as they do have a pronounced effect on functionality and long-term outcome.

## Definition of Subsyndromal Symptoms in Bipolar Disorder

In a past review, Bauer and colleagues identified 77 articles on subsyndromal BD published between 1987 and 2007, and virtually no common definition of subsyndromal mood symptoms was used (15). Until today, there is no uniform, generally accepted and operationalized definition of SSBD. Definitions can be based on being short of syndromal criteria for a major mood episode as defined by Diagnostic and Statistical Manual, 4^th^ edition (DSM IV), e.g., exhibiting at least two, but less than five criteria for a major depressive episode (MDE) (“minor depression”) (16). For subsyndromal BD II depression, Benazzi (17) proposed quite restrictive criteria taking also functionality into account: (a) residual depressive symptoms between the last two consecutive MDEs (the DSM-IV longitudinal course specifier “without full interepisode recovery”), (b) residual depressive symptoms lasting more than 2 years, (c) 2–4 MDE symptoms (recorded as present or absent with the Structured Clinical Interview for DSM IV Disorders – Clinician Version (SCID-CV), (d) mild to moderate impairment of functioning [Global Assessment of Functioning (GAF) score 60–70]. The definition by Benazzi et al., however, targets merely subsyndromal depression, but not mania or other domains such as cognition. More frequently, symptomatic rating scales such as the Young Mania Rating scale (YMRS), Mania rating Scale (MRS), Hamilton Depression Rating Scale (HDRS), or Montgomery-Asberg Depression Rating scale (MADRS) (18–20) are used to define both manic and depressive subsyndromal states. The International Society of Bipolar Disorder (ISBD) consensus criteria defines bipolar patients with a YMRS Score 8–14 as subsyndromal manic, and with a HRDS score 8–14 as subsyndromal depressed (21), but other authors allow for a wider range (YMRS 11–20, HRDS 7–17 (20). Finally, some publications equate subsyndromal states with “mild mania (YMRS 5–10)” or “mild depression (HAM-D 7–15) (22).

## Frequency of Subsyndromal Symptoms in Bipolar Disorder

A Spanish cohort study over 5 years using ISBD criteria found SSBD in more than 20% of BD patients (23). Applying in another analysis of this study, the wider criteria of De Dios et al. (20) instead of the ISBD criteria increases the percentage of bipolar spectrum patients with subsyndromal symptoms by one-third (36% vs. 22.6%) with a clear preponderance of subsyndromal depression, independent from criteria used (19). Similar figures have been reported from other observational studies (2).

Emerging subsyndromal symptoms are even more frequent in the built-up of an acute episode. Seventy-six percent of BD patients reported subsyndromal, prodromal hypomanic symptoms, and 39% subsyndromal, prodromal depressive symptoms preceding an acute episode (24). Even higher numbers have been reported by Keitner et al. (25) in a BD I cohort study: 78% of the patients reported prodromal depressive symptoms and 87% reported prodromal manic symptoms, and more than half of the patients still exhibits residual symptoms after an acute episode (54% following an MDE and 68% after a manic episode).

However, we have to keep in mind that the definition of subsyndromal states and time criteria vary between studies. Especially, the issue of duration, i.e., how long should symptoms last to satisfy SSBD diagnosis, is critical. SSBD can be rather short if a prodromal state, and lasting in the aftermath of an acute episode. SSBD can be a cross-sectional as well as a longitudinal description of a mood state; cross-sectional if based on rating scale scores at time of examination, e.g., the ISBD criteria (21), short longitudinal if longer than 1 week (22), or lasting longer than 2 years as proposed by Benazzi et al. (17).

Residual depressive symptoms are also abundant in BD II patients, 44.9% of patients have residual symptoms after and MDE despite using restrictive criteria (17). Comparing subsyndromal depressive symptoms in remitted unipolar and bipolar patients, it appears that unipolar patients may have more residual symptoms than bipolar patients, particularly in items related to anxiety and somatic complaints (26).

But even patients who are in remission according to the Clinical Global Impression scale (CGI) are not necessarily free of symptoms and impairment, and are trapped in the gap between remission and recovery. In selected clinical samples receiving optimized treatment, such as the Stanley Foundation Bipolar Network (SFBN) (10) cohort, enduring subsyndromal symptoms, especially of depression, anxiety or physical discomfort, are observable in at least 10% of bipolar patients despite fulfilling formalized criteria of remission (11).

## Impact on Functionality

SSBD is not only a predictor of early relapse (27) but also of frequent comorbidities (22), poor functional outcome and low QoL (26, 28–30). Even after a single manic episode, only one out of three patients regains psychosocial functioning at 1-year follow-up (31), suggesting that functional outcomes in BD are impaired from the very beginning. Unfortunately, there is no uniform consensus how to measure psychosocial functioning in BD. The Task Force of the ISBD examined different definitions of psychosocial functioning but without reaching a consensus (21). The task force referred to the definition provided by the International Classification of Functioning, Disability and Health (ICF) (32) in which functioning comprises three different components: body structures and functions; activities and participation; and personal environmental factors. Moreover, the authors of these guidelines underlined that this construct is probably too complex to be applied to BD, and that besides the ICF, the Functioning Assessment Short Test (FAST) scale (33) might constitute a more practical approach to measure functioning. Another widely used instruments to estimate global functioning is the GAF (34). However, the DSM-5 no longer encourages the use of the GAF. Instead, the use of the self-rated World Health Organization Disability Assessment Schedule 2.0 (WHODAS 2.0) has been recommended (35), but to date little experience exists about its usefulness and applicability in BD.

A SFBN study prospectively evaluated the association between the presence of subsyndromal depressive symptoms in 759 patients with BD and role functioning (28). Subsyndromal depression was operationalized using cut-off scores on the Inventory of Depressive Symptoms-Clinician Rated (IDS-C) and the CGI for Bipolar Disorder (CGI-BP). Patients were divided into three groups: no depression (n = 292, IDS-C score of <13 for the prior 2 weeks), subsyndromal depression (n = 291, IDS-C scores 13 to 27 for the prior 2 weeks), and syndromal depression (n = 179, IDS-C scores > 28 for the prior 2 weeks). To ensure that all subjects were not currently experiencing depressive symptoms as part of a mixed mania, a score of 1 (not ill) on the CGI-BP severity of mania item was also mandatory. Patient functioning in four role domains (work, home duties, family life, and friendships) was assessed using the Life Functioning Questionnaire (LFQ), a 5-min, 14-item, gender-neutral self-report questionnaire designed to assess role function over the preceding month (36). The subsyndromal depressed group was significantly more likely than the no depression group to report impairment at work (64% vs. 31%), with duties at home (75% vs. 38%), in their relationships with family (59% vs. 34%) and friends (56% vs. 18%), and in life functioning overall (70% vs. 32%), and across all domains of role function, the proportion of patients who were impaired were more similar in the subsyndromal and syndromal depression groups than to the no depression group. But not only persistent low mood determines functionality, but also unstable affect, both negative and positive. A study in 27 subjects with BD I examining the prospective influence of inter-episode affect dysregulation on symptoms and functional impairment, and found that mood instability in general during the inter-episode period was associated with greater impairment in home and work functioning (37).

The OPTHYMUM study (29) in 525 BD patients across France chose the converse approach. This cross-sectional study looked into the associations and consequences of low functioning, defined as a GAF score <60. These “low functioning patients” had significantly more frequent emotional subsyndromal symptoms (emotional lability and numbing), disruption of circadian rhythms, sexual disorders, and perceived more cognitive deficits. In addition, they suffered more social and family stigma, were more frequently unemployed and had increased numbers of manic episodes and psychotic symptoms.

The tight association between subsyndromal symptoms and low GAF scores has also been confirmed by McQueen et al. (22) using prospectively collected life charting data from 138 patients with BD. Looking into co-morbidities, SSBD patients were on an even higher risk of eating and anxiety disorders than syndromal patients. A *post hoc* analysis of prospectively collected data by Bennett et al. demonstrated in the SFBN cohort a significant interaction between attention-deficit and anxiety comorbidity and low GAF scores (38). In line with this, a prospective Dutch cohort study demonstrated that subsyndromal anxiety also been impacts on functionality in unipolar depression (39).

In youth with BD-I, a relatively long, predominantly slow-onset mania prodrome appears to be common, including subthreshold manic and depressive psychopathology symptoms (40). Little, however, has been reported about functional prodromal symptoms before a first manic/mixed episode and the diagnosis of a BD. A literature search by Faedda et al. prospectively identified subsyndromal symptoms both of mania and depression as precursors of BD that typically arose years prior to syndromal onset (14). Based on a survey conducted in members of the NDMDA, Hirschfeld and colleagues noted that in majority of patients many domains of social functioning had been already dysfunctional prior to a first BD episode (41), most likely due to subsyndromal symptoms.

In a cross-sectional study of Keitner et al. (25) more than half of the patients disclosed residual symptoms of depression (54%) and mania (68%). However, cognitive symptoms were consistently the most common symptoms reported by patients across studies, and constitute one of the three determinants of functional outcome in the model by Samalin et al. (5). Even when euthymic, compared with people without mood disorders, people with BD have cognitive impairment (42). This may, at least in part, explain why previously able people decline in their functioning (43). In recent years, more sophisticated research has been conducted to objectify the subjective reports of cognitive decline. In the domains of cognition—executive function, attention, processing speed, verbal memory and visual memory—learning memory and executive function may be more impaired than others (44–48). Looking cross-sectionally at psychosocial and occupational function, impaired cognition has a significant effect on both outcomes, whereas residual depressive symptoms seem to impact mainly on psychosocial capabilities (49).

## Impact on Quality of Life

QoL is a broad construct taken to represent aspects of functioning and satisfaction in occupational, environmental, social, physical, and psychological aspects of life (50).QoL has not only become an increasingly important outcome parameter in clinical trials, but also a target for web-based psychoeducational self-monitoring programs (51, 52). Different scales exist to make the rather holistic and fuzzy defined term “Quality of life” measurable (53). Especially for BD, Michalak and colleagues developed the QoL in Bipolar Disorder (QoL.BD) scale. It comprises 56 items rated from 1 to 5, evaluating the domains physical, sleep, mood, cognition, leisure, social, spirituality, finances, household, self-esteem, independence, identity, work, and education. A global score is obtained with higher scores indicating a better QoL, and score below 170 indicates poor QoL (54). A meta-analysis of 66 studies demonstrated significant differences in QoL outcomes between euthymic BD patients and healthy controls with lower QoL in the euthymic patients (55). In a cross-sectional study in 60 clinically stable Bipolar I outpatients with only mild residual symptoms, QoL correlated significantly with resilience, internalized stigma, and, again, residual symptoms of depression. No significant correlations were observed between QoL and residual manic symptoms (56).

The detrimental impact of comorbid anxiety disorders fulfilling full diagnostic criteria on BD has been well established (57). The interaction between anxiety and mood symptoms on a subsyndromal level is still poorly understood and further research is demanded. The few data available suggest that also subsyndromal anxiety has a marked impact on QoL in euthymic bipolar patients. In a cross-sectional Mexican study using the QoL.BD scale and a score of 170 as cut-off for poor QoL, anxious symptoms affected the perceived QoL more than subthreshold symptoms of mania and depression and more than other variables related to the course of BD, such as number of hospitalizations, and even a comorbid diagnosis of full-criteria generalized anxiety disorder (GAD). Of note, subthreshold manic (not depressive) symptoms were in this study the second parameter related to poor QoL (58). **Table 1** summarizes the proven or assumed impact of different subsyndromal symptoms on functionality and QoL. As a limitation, the reader should note that the table reflects the authors’ personal views based on the results of their literature search. A full systematic review, and possibly a meta-analytic processing of the results, would be needed and desirable to validate these findings.

**Table 1:** Summary of the proposed impact of the different domains of SSBD following an acute episode on functionality and QoL.

Domain of SSBD	Impact on functionality	Impact on QoL
Depression	+++	++
Mania	++	++
Cognition	+++	(+)
Disturbed circadian rhythm	++	+
Psychosis	(+)	?
Comorbid anxiety	++	+++

Note that the table reflects the author’s personal view based on the literature cited in this review.

+++, marked impact; ++, moderate impact; +, mild impact; (+), possible impact; ?, unknown.

But also, somatic malaise has a clear impact on QoL. A large Spanish cohort study using also a cross-sectional design demonstrated that gastrointestinal and somatic symptoms, as well as genital symptoms occur more frequently in SSBD than in the general population (26).

## Treatment

Whereas only few treatment studies have targeted SSBD so far, there is some consensus to use similar guidance as for treatment-resistant full blown episodes to eradicate subsyndromal symptoms (59, 60). This includes checking whether the diagnosis is correct, excluding (or treating) psychiatric co-morbidities such as addiction, anxiety and personality disorders, optimization of the medication dose including therapeutic drug monitoring, augmentation and combination strategies, experimental treatments such as ketamine infusion, considering physical treatments such as electroconvulsive therapy, repetitive transcranial magnetic stimulation, or sleep phase advance protocols, reviewing the effectiveness of ongoing psychotherapy, minimizing or eliminating social and occupational distress, and a careful check-up of physical health as organic factors might contribute to persistent symptoms. Some subsyndromal symptoms like anxiety might be targeted with specific drugs normally not used for bipolar core symptoms (61, 62); however, there is some controversy about this approach. The symptom-oriented treatment approach to treat anxiety symptoms with anxiolytics or anti-anxiety drugs has been questioned by the results of a study of Bauer et al. (63). Using naturalistic data, Bauer and co-workers investigated retrospectively the relationship between the use of adjunctive anxiolytics and the time spent in episodes or with SSBD in a *post hoc* analysis of 310 patients with BD. Patients with BD who were taking adjunctive anxiolytics spent significantly more time ill. The authors concluded that, while the study design cannot determine causality, there is an obvious need for controlled studies of a possibly detrimental impact of adjunctive medications for anxiety on the course of BD.

For the prevention and control of subsyndromal symptoms and, by this, preventing relapse lithium levels in the high therapeutic range (0.8–1.0 mmol/l) had been more effective than low-range lithium levels (0.4–0.6 mmol/l). Patients with low-range levels had 2.6 times the risk of major affective relapse as those given lithium for high-range levels, and nearly twice the risk of developing subsyndromal symptoms (24).

The prominent role of lithium in controlling prodromal symptoms of emerging mania or depression has also been confirmed by a *post hoc* analysis of two controlled maintenance studies comparing lithium, lamotrigine, and placebo (64). Compared to placebo, both lithium and lamotrigine treatment significantly delayed emerging subsyndromal symptoms of either polarity and prolongated time from first subsyndromal symptoms to intervention for a full-blown episode. Further analysis showed that this was by large due to suppressing subsyndromal manic or mixed symptoms. On the other hand, lamotrigine appeared to be effective in delaying time from first subsyndromal depressive symptoms to a mood episode (18). The risk of emerging subsyndromal depression or of a MDE in lithium-treated patients might be related to changes in thyroid function. Those lithium-treated BD I patients who needed intervention for depression in these studies had a significantly higher adjusted mean TSH level than those who did not (65).

In the aftermath of an acute episode, switching from different atypical antipsychotics to aripiprazole has been reported to improve subsyndromal symptoms in an observational 24-week study (66). In part, this may also be explained by aripiprazole’s lack of anticholinergic effects and its potential antidepressant effects, thus reducing possible medication-induced cognitive and affective impairment.

Specially to overcome functional impairment, cognitive-behavioral, family focused, or social-rhythm–based psychotherapies have been shown to be effective alongside optimized pharmacological treatment (67–69). In a small study with children and adolescents, both omega-3 fatty acid supplementation (Omega3) and individual family psychoeducational psychotherapy (IF-PEP) showed medium-size effects on subsyndromal depression (70). Accumulating evidence suggest that functional remediation may have good potential to boost the recovery process in bipolar patients with subsyndromal symptoms and might be not only more effective than treatment as usual, but also than psychoeducation (71, 72). Case reports suggested that also Eye Movement Desensitization and Reprocessing (EMDR) may positively affect residual bipolar symptoms (73, 74). More recently, a small, 12-week pilot study in patients with SSBD and post-traumatic stress disorder demonstrated that EMDR has significant effects on subsyndromal manic symptoms, and to a lesser degree on subsyndromal depression (75).

## Discussion

As a fair estimate, SSBD affects between 20 and 50% of bipolar patients, depending on the definition applied. Especially subsyndromal depression interferes with role functioning in essential domains of normal life, such as work, duties at home and maintaining relationships. Besides residual depression, enduring cognitive impairment in a variety of domains determines psychosocial and occupational outcome. Subsyndromal depression and cognitive decline in SSBD have also been identified as two out of three main driver of low function in a structural equation model as described by Samalin and coworkers, the third one being sleep deprivation (5). Except of one study (58), subsyndromal mania, however, appears not as a main driver of low QoL and functioning, but has been much less investigated than subsyndromal depression. As far as QoL is concerned, anxiety, ranging from just symptoms to full blown comorbid disorder, and physical malaise appear to be inversely related to QoL in BD, including SSBD. However, the potential pathways that might mediate the observed relationships between SSBD and functionality and QoL are still speculative and need further investigation. But in summary, the marked impact of SSBD both on psycho-social functioning and QoL is obvious and well documented in a fair number of studies.

This is in contrast to the relative paucity of treatment studies, which is even more true for pharmacological than for psychological approaches. This may, in part, be due to the absence of an official indication approved by regulatory authorities and, as a consequence, uncertainty about eligibility for reimbursement in some health insurance systems as patients may be categorized as “euthymic”. In addition, there is an absence of a generally accepted definition of SSBD and uniform cut-off criteria, that makes it difficult to compare between the few studies available and derive recommendations. If a patient is on lithium, optimizing lithium levels appear to ameliorate subsyndromal mood and cognitive symptoms. Switching to medication that do not add to potential cognitive impairment by anticholinergic side effects might also be a strategy to consider. Recent research, however, clearly pointed out that a tailored psychotherapy might be effective in overcoming SSBD. Especially functional and cognitive remediation seems to be effective, and emerging new techniques as EMDR might add in the future to the treatment portfolio. However, although there is some evidence emerging more recently, more research focus and effort is still needed. Most studies included and mixed both subsyndromal depression and subsyndromal hypomanic patients, without further differentiation of outcomes. More studies looking into the different dimension of SSBD (mania, depression, cognition, psychosis) separately are clearly demanded. Finally, most important, a verified and generally accepted definition of SSBD and its constituents needs to be developed to allow for randomized studies with comparable inclusion/exclusion criteria.

## Author Contributions

The authors designed the work, conducted the necessary literature search, drafted the manuscript, provide approval for publication, and agree to be accountable for all aspects of the work.

## Conflict of Interest

The authors declare that the research was conducted in the absence of any commercial or financial relationships that could be construed as a potential conflict of interest.

## References

[1] van der Markt A Klumpers UM Draisma S Dols A Nolen WA Post RM Testing a clinical staging model for bipolar disorder using longitudinal life chart data. BipolarDisord (2019) 21(3):228–34. 10.1111/bdi.12727 PMC6590317 30447123

[2] Marangell LB The importance of subsyndromal symptoms in bipolar disorder. J Clin Psychiatry (2004) 65 Suppl 10:24–7. 15242329

[3] Serafini G Vazquez GH Gonda X Pompili M Rihmer Z Amore M Depressive residual symptoms are associated with illness course characteristics in a sample of outpatients with bipolar disorder. Eur Arch Psychiatry Clin Neurosci (2018) 268(8):757–68. 10.1007/s00406-018-0875-5 29417206

[4] van Os J Kapur S Schizophrenia. Lancet (2009) 374(9690):635–45. 10.1016/S0140-6736(09)60995-8 19700006

[5] Samalin L Boyer L Murru A Pacchiarotti I Reinares M Bonnin CM Residual depressive symptoms, sleep disturbance and perceived cognitive impairment as determinants of functioning in patients with bipolar disorder. J Affect Disord (2017) 210:280–6. 10.1016/j.jad.2016.12.054 28068616

[6] Soreca I Frank E Kupfer DJ The phenomenology of bipolar disorder: what drives the high rate of medical burden and determines long-term prognosis? Depress Anxiety (2009) 26(1):73–82. 10.1002/da.20521 18828143 PMC3308337

[7] Murru A Pacchiarotti I Verdolini N Reinares M Torrent C Geoffroy PA Modifiable and non-modifiable factors associated with functional impairment during the inter-episodic periods of bipolar disorder. Eur Arch Psychiatry Clin Neurosci (2018) 268(8):749–55. 10.1007/s00406-017-0811-0 28534186

[8] Roux P Raust A Cannavo AS Aubin V Aouizerate B Azorin JM Associations between residual depressive symptoms, cognition, and functioning in patients with euthymic bipolar disorder: results from the FACE-BD cohort. Br J Psychiatry (2017) 211(6):381–7. 10.1192/bjp.bp.117.201335 29051175

[9] Strejilevich S Szmulewicz A Igoa A Marengo E Caravotta P Martino D Episodic density, subsyndromic symptoms, and mood instability in late-life bipolar disorders: A 5-year follow-up study. Int J Geriatr Psychiatry (2019) 34(7):950–6. 10.1002/gps.5094 30864181

[10] Sierra P Livianos L Arques S Castello J Rojo L Prodromal symptoms to relapse in bipolar disorder. Aust N Z J Psychiatry (2007) 41(5):385–91. 10.1080/00048670701266854 17464729

[11] Jackson A Cavanagh J Scott J Sierra P Livianos L Arques S A systematic review of manic and depressive prodromes Prodromal symptoms to relapse in bipolar disorder. J Affect Disord (2003) 74(3):209–17. 10.1016/s0165-0327(02)00266-5 12738039

[12] Wong G Lam D Jackson A Cavanagh J Scott J Sierra P The development and validation of the coping inventory for prodromes of mania A systematic review of manic and depressive prodromes Prodromal symptoms to relapse in bipolar disorder Life events and the onset of mania. J Affect Disord (1999) 53(1):57–65. 10.1016/s0165-0327(98)00096-2 10363667

[13] Parker G Predicting onset of bipolar disorder from subsyndromal symptoms: a signal question? Br J Psychiatry (2010) 196(2):87–8. 10.1192/bjp.bp.109.074898 20118448

[14] Faedda GL Marangoni C Serra G Salvatore P Sani G Vazquez GH Precursors of bipolar disorders: a systematic literature review of prospective studies. J ClinPsychiatry (2015) 76(5):614–24. 10.4088/JCP.13r08900 26035191

[15] Bauer M Glenn T Grof P Schmid R Pfennig A Whybrow PC Subsyndromal mood symptoms: a useful concept for maintenance studies of bipolar disorder? Psychopathology (2010) 43(1):1–7. 10.1159/000255957 19893338

[16] Marangell LB Dennehy EB Miyahara S Wisniewski SR Bauer MS Rapaport MH The functional impact of subsyndromal depressive symptoms in bipolar disorder: data from STEP-BD. JAffectDisord (2009) 114(1-3):58–67. 10.1016/j.jad.2008.07.006 PMC2704060 18708263

[17] Benazzi F Prevalence and clinical correlates of residual depressive symptoms in bipolar II disorder. PsychotherPsychosom (2001) 70(5):232–8. 10.1159/000056260 11509892

[18] Frye MA Yatham LN Calabrese JR Bowden CL Ketter TA Suppes T Incidence and time course of subsyndromal symptoms in patients with bipolar I disorder: an evaluation of 2 placebo-controlled maintenance trials. J ClinPsychiatry (2006) 67(11):1721–8. 10.4088/JCP.v67n1108 17196051

[19] De Dios C Agud JL Ezquiaga E Garcia-Lopez A Soler B Vieta E Syndromal and subsyndromal illness status and five-year morbidity using criteria of the International Society for Bipolar Disorders compared to alternative criteria. Psychopathology (2012) 45(2):102–8. 10.1159/000329740 22269982

[20] De Dios C Ezquiaga E Garcia A Soler B Vieta E Time spent with symptoms in a cohort of bipolar disorder outpatients in Spain: a prospective, 18-month follow-up study. JAffectDisord (2010) 125(1-3):74–81. 10.1016/j.jad.2009.12.006 20034673

[21] Tohen M Frank E Bowden CL Colom F Ghaemi SN Yatham LN The International Society for Bipolar Disorders (ISBD) Task Force report on the nomenclature of course and outcome in bipolar disorders. Bipolar Disord (2009) 11(5):453–73. 10.1111/j.1399-5618.2009.00726.x 19624385

[22] MacQueen GM Marriott M Begin H Robb J Joffe RT Young LT Subsyndromal symptoms assessed in longitudinal, prospective follow-up of a cohort of patients with bipolar disorder. BipolarDisord (2003) 5(5):349–55. 10.1034/j.1399-5618.2003.00048.x 14525555

[23] De Dios C Ezquiaga E Agud JL Vieta E Soler B Garcia-Lopez A Subthreshold symptoms and time to relapse/recurrence in a community cohort of bipolar disorder outpatients. JAffectDisord (2012) 143(1-3):160–5. 10.1016/j.jad.2012.05.047 22925351

[24] Keller MB Lavori PW Kane JM Gelenberg AJ Rosenbaum JF Walzer EA Subsyndromal symptoms in bipolar disorder. A comparison of standard and low serum levels of lithium. ArchGenPsychiatry (1992) 49(5):371–6. 10.1001/archpsyc.1992.01820050035005 1586272

[25] Keitner GI Solomon DA Ryan CE Miller IW Mallinger A Kupfer DJ Prodromal and residual symptoms in bipolar I disorder. Compr Psychiatry (1996) 37(5):362–7. 10.1016/s0010-440x(96)90018-8 8879911

[26] Vieta E Sanchez-Moreno J Lahuerta J Zaragoza S Subsyndromal depressive symptoms in patients with bipolar and unipolar disorder during clinical remission. JAffectDisord (2008) 107(1-3):169–74. 10.1016/j.jad.2007.08.007 17870184

[27] Radua J Grunze H Amann BL Meta-Analysis of the Risk of Subsequent Mood Episodes in Bipolar Disorder. PsychotherPsychosom (2017) 86(2):90–8. 10.1159/000449417 28183076

[28] Altshuler LL Post RM Black DO Keck PE Jr. Nolen WA Frye MA Subsyndromal depressive symptoms are associated with functional impairment in patients with bipolar disorder: results of a large, multisite study. J Clin Psychiatry (2006) 67(10):1551–60. 10.4088/JCP.v67n1009 17107246

[29] Samalin L Llorca PM Giordana B Milhiet V Yon L El-Hage W Residual symptoms and functional performance in a large sample of euthymic bipolar patients in France (the OPTHYMUM study). J AffectDisord (2014) 159:94–102. 10.1016/j.jad.2014.02.023 24679396

[30] Bauer M Glenn T Grof P Rasgon NL Marsh W Sagduyu K Frequency of subsyndromal symptoms and employment status in patients with bipolar disorder. SOCPSYCHIATRY PSYCHIATREPIDEMIOL (2009) 44(7):515–22. 10.1007/s00127-008-0464-4 19011720

[31] Tohen M Hennen J Zarate CM Baldessarini RJ Strakowski SM Stoll AL Two-year syndromal and functional recovery in 219 cases of first-episode major affective disorder with psychotic features. AmJ Psychiatry (2000) 157(2):220–8. 10.1176/appi.ajp.157.2.220 10671390

[32] Organization WH International classification of functioning, disability and health : ICF. Geneva: World Health Organization (2001).

[33] Rosa AR Sanchez-Moreno J Martinez-Aran A Salamero M Torrent C Reinares M Validity and reliability of the Functioning Assessment Short Test (FAST) in bipolar disorder. ClinPractEpidemiolMentHealth (2007) 3:5. 10.1186/1745-0179-3-5 PMC1904447 17555558

[34] American Psychiatric A Diagnostic and statistical manual of mental disorders. Washington DC: APA Press (1987).

[35] Ustun TB Chatterji S Kostanjsek N Rehm J Kennedy C Epping-Jordan J Developing the World Health Organization Disability Assessment Schedule 2.0. Bull World Health Organ (2010) 88(11):815–23. 10.2471/BLT.09.067231 PMC2971503 21076562

[36] Altshuler L Mintz J Leight K The Life Functioning Questionnaire (LFQ): a brief, gender-neutral scale assessing functional outcome. Psychiatry Res (2002) 112(2):161–82. 10.1016/s0165-1781(02)00180-4 12429362

[37] Gershon A Eidelman P Inter-episode affective intensity and instability: predictors of depression and functional impairment in bipolar disorder. J Behav Ther Exp Psychiatry (2015) 46:14–8. 10.1016/j.jbtep.2014.07.005 PMC4254202 25164093

[38] Bennett F Hodgetts S Altshuler L Close A Frye M Grunze H Predictors of Psychosocial Outcome of Bipolar Disorder: Results from the Stanley Foundation Bipolar Network. Int J Bipolar Disord (2019) 7:8. 10.1186/s40345-019-0169-5 31840207 PMC6911815

[39] Karsten J Penninx BWJH Verboom CE Nolen WA Hartman CA Course and risk factors of functional impairment in subtreshold depression and anxiety. Depression Anxiety (2013) 30(4):386–94. 10.1002/da.22021 23165799

[40] Correll CU Hauser M Penzner JB Auther AM Kafantaris V Saito E Type and duration of subsyndromal symptoms in youth with bipolar I disorder prior to their first manic episode. Bipolar Disord (2014) 16(5):478–92. 10.1111/bdi.12194 PMC4186919 24597782

[41] Hirschfeld RM Lewis L Vornik LA Perceptions and impact of bipolar disorder: how far have we really come? Results of the national depressive and manic-depressive association 2000 survey of individuals with bipolar disorder. J Clin Psychiatry (2003) 64(2):161–74. 10.4088/JCP.v64n0209 12633125

[42] Martinez-Aran A Vieta E Colom F Torrent C Sanchez-Moreno J Reinares M Cognitive impairment in euthymic bipolar patients: implications for clinical and functional outcome. Bipolar Disord (2004) 6(3):224–32. 10.1111/j.1399-5618.2004.00111.x 15117401

[43] Rubinsztein JS Michael A Paykel ES Sahakian BJ Cognitive impairment in remission in bipolar affective disorder. PsycholMed (2000) 30(5):1025–36. 10.1017/S0033291799002664 12027040

[44] Sumiyoshi T Toyomaki A Kawano N Kitajima T Kusumi I Ozaki N Verbal Memory Impairment in Patients with Subsyndromal Bipolar Disorder. Front Psychiatry (2017) 8:168. 10.3389/fpsyt.2017.00168 28966598 PMC5605624

[45] Martinez-Aran A Vieta E Torrent C Sanchez-Moreno J Goikolea JM Salamero M Functional outcome in bipolar disorder: the role of clinical and cognitive factors. Bipolar Disord (2007) 9(1-2):103–13. 10.1111/j.1399-5618.2007.00327.x 17391354

[46] Bonnin CM Martinez-Aran A Torrent C Pacchiarotti I Rosa AR Franco C Clinical and neurocognitive predictors of functional outcome in bipolar euthymic patients: a long-term, follow-up study. JAffectDisord (2010) 121(1-2):156–60. 10.1016/j.jad.2009.05.014 19505727

[47] Torres IJ DeFreitas CM DeFreitas VG Bond DJ Kunz M Honer WG Relationship between cognitive functioning and 6-month clinical and functional outcome in patients with first manic episode bipolar I disorder. Psychol Med (2011) 41(5):971–82. 10.1017/s0033291710001613 20810001

[48] Jimenez-Lopez E Sanchez-Morla EM Aparicio AI Lopez-Villarreal A Martinez-Vizcaino V Rodriguez-Jimenez R Psychosocial functioning in patients with psychotic and non-psychotic bipolar I disorder. A comparative study with individuals with schizophrenia. J Affect Disord (2018) 229:177–85. 10.1016/j.jad.2017.12.094 29316520

[49] Mur M Portella MJ Martinez-Aran A Pifarre J Vieta E Influence of clinical and neuropsychological variables on the psychosocial and occupational outcome of remitted bipolar patients. Psychopathology (2009) 42(3):148–56. 10.1159/000207456 19276630

[50] Morton E Michalak EE Murray G What does quality of life refer to in bipolar disorders research? A systematic review of the construct’s definition, usage and measurement. J Affect Disord (2017) 212:128–37. 10.1016/j.jad.2017.01.026 28160685

[51] Fletcher K Foley F Murray G Web-Based Self-Management Programs for Bipolar Disorder: Insights From the Online, Recovery-Oriented Bipolar Individualised Tool Project. J MedInternetRes (2018) 20(10):e11160. 10.2196/11160 PMC6231852 30355553

[52] Morton E Hole R Murray G Buzwell S Michalak E Experiences of a Web-Based Quality of Life Self-Monitoring Tool for Individuals With Bipolar Disorder: A Qualitative Exploration. JMIR Ment Health (2019) 6(12):e16121. 10.2196/16121 31799936 PMC6920912

[53] Murray G Michalak EE The quality of life construct in bipolar disorder research and practice: past, present, and possible futures. Bipolar Disord (2012) 14(8):793–6. 10.1111/bdi.12016 23131090

[54] Michalak EE Murray G Development of the QoL.BD: a disorder-specific scale to assess quality of life in bipolar disorder. Bipolar Disord (2010) 12(7):727–40. 10.1111/j.1399-5618.2010.00865.x 21040290

[55] Pascual-Sánchez A Jenaro C Montes-Rodríguez JM Quality of life in euthymic bipolar patients: A systematic review and meta-analysis. J Affect Disord (2019) 255:105–15. 10.1016/j.jad.2019.05.032 31150940

[56] Post F Pardeller S Frajo-Apor B Kemmler G Sondermann C Hausmann A Quality of life in stabilized outpatients with bipolar I disorder: Associations with resilience, internalized stigma, and residual symptoms. J Affect Disord (2018) 238:399–404. 10.1016/j.jad.2018.05.055 29909303

[57] Yapici Eser H Kacar AS Kilciksiz CM Yalçinay-Inan M Ongur D Prevalence and Associated Features of Anxiety Disorder Comorbidity in Bipolar Disorder: A Meta-Analysis and Meta-Regression Study. Front Psychiatry (2018) 9:229. 10.3389/fpsyt.2018.00229 29997527 PMC6030835

[58] Yoldi-Negrete M Morera D Palacios-Cruz L Camarena B Ortega H Castañeda-Franco M Subsyndromal anxiety: Does it affect the quality of life? A study on euthymic patients with bipolar disorder. Eur J Psychiatry (2019) 33(4):159–64. 10.1016/j.ejpsy.2019.06.005

[59] Hidalgo-Mazzei D Berk M Cipriani A Cleare AJ Florio AD Dietch D Treatment-resistant and multi-therapy-resistant criteria for bipolar depression: consensus definition. BrJPsychiatry (2019) 214(1):27–35. 10.1192/bjp.2018.257 PMC7613090 30520709

[60] Fountoulakis KN Refractoriness in bipolar disorder: definitions and evidence-based treatment. CNSNeurosciTher (2012) 18(3):227–37. 10.1111/j.1755-5949.2011.00259.x PMC6493614 22070611

[61] Bernhard B Born C Seemüller F Grunze H Levetiracetam add-on treatment for bipolar patients suffering from subsyndromal symptoms: preliminary data of an open 6-month longitudinal study. Eur Neuropsychopharmacol (2005) 15 Suppl 3:416. 10.1016/S0924-977X(05)80844-2

[62] Vieta E Martinez-Aran A Nieto E Colom F Reinares M Benabarre A Adjunctive gabapentin treatment of bipolar disorder. EurPsychiatry (2000) 15(7):433–7. 10.1016/S0924-9338(00)00514-9 11112936

[63] Bauer M Glenn T Grof P Rasgon NL Marsh W Sagduyu K Relationship between adjunctive medications for anxiety and time spent ill in patients with bipolar disorder. IntJPsychiatry ClinPract (2009) 13(1):70–7. 10.1080/13651500802450514 24946124

[64] Goodwin GM Bowden CL Calabrese JR Grunze H Kasper S White R A pooled analysis of 2 placebo-controlled 18-month trials of lamotrigine and lithium maintenance in bipolar I disorder. J Clin Psychiatry (2004) 65(3):432–41. 10.4088/JCP.v65n0321 15096085

[65] Frye MA Yatham L Ketter TA Goldberg J Suppes T Calabrese JR Depressive relapse during lithium treatment associated with increased serum thyroid-stimulating hormone: results from two placebo-controlled bipolar I maintenance studies. Acta PsychiatrScand (2009) 120(1):10–3. 10.1111/j.1600-0447.2008.01343.x 19183414

[66] Woo YS Bahk WM Park YM Chung S Yoon BH Won S Effects of switching to aripiprazole from current atypical antipsychotics on subsyndromal symptoms and tolerability in patients with bipolar disorder. Int Clin Psychopharmacol (2016) 31(5):275–86. 10.1097/yic.0000000000000136 27487259

[67] Miklowitz DJ Otto MW Frank E Reilly-Harrington NA Wisniewski SR Kogan JN Psychosocial treatments for bipolar depression: a 1-year randomized trial from the Systematic Treatment Enhancement Program. ArchGenPsychiatry (2007) 64(4):419–26. 10.1001/archpsyc.64.4.419 PMC3579612 17404119

[68] Gonzalez-Isasi A Echeburua E Liminana JM Gonzalez-Pinto A Predictors of good outcome in patients with refractory bipolar disorder after a drug or a drug and cognitive-behavioral treatment. ComprPsychiatry (2012) 53(3):224–9. 10.1016/j.comppsych.2011.05.001 21658693

[69] Miklowitz DJ Otto MW Frank E Reilly-Harrington NA Kogan JN Sachs GS Intensive psychosocial intervention enhances functioning in patients with bipolar depression: results from a 9-month randomized controlled trial. AmJ Psychiatry (2007) 164(9):1340–7. 10.1176/appi.ajp.2007.07020311 PMC3579578 17728418

[70] Fristad MA Young AS Vesco AT Nader ES Healy KZ Gardner W A Randomized Controlled Trial of Individual Family Psychoeducational Psychotherapy and Omega-3 Fatty Acids in Youth with Subsyndromal Bipolar Disorder. J Child Adolesc Psychopharmacol (2015) 25(10):764–74. 10.1089/cap.2015.0132 PMC4691654 26682997

[71] Sole B Bonnin CM Mayoral M Amann BL Torres I Gonzalez-Pinto A Functional remediation for patients with bipolar II disorder: improvement of functioning and subsyndromal symptoms. Eur Neuropsychopharmacol (2015) 25(2):257–64. 10.1016/j.euroneuro.2014.05.010 24906790

[72] Reinares M Sanchez-Moreno J Fountoulakis KN Psychosocial interventions in bipolar disorder: what, for whom, and when. JAffectDisord (2014) 156:46–55. 10.1016/j.jad.2013.12.017 24439829

[73] Oh D Kim D Eye movement desensitization and reprocessing for posttraumatic stress disorder in bipolar disorder. Psychiatry Invest (2014) 11(3):340–1. 10.4306/pi.2014.11.3.340 PMC4124196 25110510

[74] Landin-Romero R Novo P Vicens V McKenna PJ Santed A Pomarol-Clotet E EMDR therapy modulates the default mode network in a subsyndromal, traumatized bipolar patient. Neuropsychobiology (2013) 67(3):181–4. 10.4306/pi.2014.11.3.340 23548794

[75] Novo P Landin-Romero R Radua J Vicens V Fernandez I Garcia F Eye movement desensitization and reprocessing therapy in subsyndromal bipolar patients with a history of traumatic events: a randomized, controlled pilot-study. Psychiatry Res (2014) 219(1):122–8. 10.1016/j.psychres.2014.05.012 24880581

